# Publisher’s Note: We Changed Page Numbers to Article Numbers for Articles Published in *Journal of Xenobiotics* Volumes 1–9

**DOI:** 10.3390/jox12010005

**Published:** 2022-03-03

**Authors:** Journal of Xenobiotics Editorial Office

**Affiliations:** MDPI AG, St. Alban-Anlage 66, 4052 Basel, Switzerland; jox@mdpi.com

The *Journal of Xenobiotics* [1] was published by PAGEPress from Volume 1 (2011) to Volume 9 (2019). Since Volume 10, Issue 1 (2020), the *Journal of Xenobiotics* has been published by MDPI.

Previous articles in Volumes 1–9, published by PAGEPress in open access under a CC-BY (or CC-BY-NC-ND) license, are now hosted by MDPI on mdpi.com as a courtesy and upon agreement with PAGEPress.

To standardize the metadata format of all previous articles, MDPI republished 92 articles in Volumes 1–9 with article numbers replacing page numbers (Table 1). MDPI also corrected Issue Number errors that occurred during importing of batch data for 14 articles in Volume 3 (Table 2).

## Figures and Tables

**Table 1:** MDPI changed page numbers to articles numbers for 92 articles in Volumes 1–9.

DOI	Previous Page Number	Current Article Number
10.4081/xeno.2011.e1	1–5	e1
10.4081/xeno.2011.e2	6–8	e2
10.4081/xeno.2011.e3	9–16	e3
10.4081/xeno.2011.e4	17–21	e4
10.4081/xeno.2011.e6	29–34	e6
10.4081/xeno.2011.e5	22–28	e5
10.4081/xeno.2011.e7	35–37	e7
10.4081/xeno.2011.e8	38–44	e8
10.4081/xeno.2012.e1	1–6	e1
10.4081/xeno.2012.e2	7–12	e2
10.4081/xeno.2012.e3	13–17	e3
10.4081/xeno.2012.e4	18–23	e4
10.4081/xeno.2012.e5	24–27	e5
10.4081/xeno.2012.e6	28–34	e6
10.4081/xeno.2012.e7	35–40	e7
10.4081/xeno.2012.e8	41–42	e8
10.4081/xeno.2012.e9	43–46	e9
10.4081/xeno.2012.e10	47–52	e10
10.4081/xeno.2013.e1	1–8	e1
10.4081/xeno.2013.e2	9–13	e2
10.4081/xeno.2013.e3	14–23	e3
10.4081/xeno.2013.e4	19–23	e4
10.4081/xeno.2013.e5	24–28	e5
10.4081/xeno.2013.e6	29–35	e6
10.4081/xeno.2013.e7	36–39	e7
10.4081/xeno.2013.e8	40–48	e8
10.4081/xeno.2013.s1.e1	1–2	e1
10.4081/xeno.2013.s1.e2	3–5	e2
10.4081/xeno.2013.s1.e3	6–7	e3
10.4081/xeno.2013.s1.e4	8–10	e4
10.4081/xeno.2013.s1.e5	11–13	e5
10.4081/xeno.2013.s1.e6	14–16	e6
10.4081/xeno.2013.s1.e7	17–19	e7
10.4081/xeno.2013.s1.e8	20–22	e8
10.4081/xeno.2013.s1.e9	23–25	e9
10.4081/xeno.2013.s1.e10	26–28	e10
10.4081/xeno.2013.s1.e11	29–30	e11
10.4081/xeno.2013.s1.e12	31–33	e12
10.4081/xeno.2013.s1.e13	34–35	e13
10.4081/xeno.2013.s1.e14	36–39	e14
10.4081/xeno.2014.1991	1–7	1991
10.4081/xeno.2014.2240	8–13	2240
10.4081/xeno.2014.2264	20–28	2264
10.4081/xeno.2014.2272	14–19	2272
10.4081/xeno.2014.3945	29–39	3945
10.4081/xeno.2014.4596	40–45	4596
10.4081/xeno.2014.4680	46–52	4680
10.4081/xeno.2014.4891	53–55	4891
10.4081/xeno.2014.4892	56–58	4892
10.4081/xeno.2014.4893	59–61	4893
10.4081/xeno.2014.4894	62–64	4894
10.4081/xeno.2014.4895	65–67	4895
10.4081/xeno.2014.4896	68–69	4896
10.4081/xeno.2014.4897	70–72	4897
10.4081/xeno.2014.4898	73–75	4898
10.4081/xeno.2014.4899	76–77	4899
10.4081/xeno.2014.4900	78–80	4900
10.4081/xeno.2014.4901	81–84	4901
10.4081/xeno.2014.4902	85–87	4902
10.4081/xeno.2014.4903	88–91	4903
10.4081/xeno.2014.4904	92–93	4904
10.4081/xeno.2014.4905	94–96	4905
10.4081/xeno.2015.5086	8–14	5086
10.4081/xeno.2015.5125	1–7	5125
10.4081/xeno.2015.5264	15–19	5264
10.4081/xeno.2015.5369	20–23	5369
10.4081/xeno.2015.5769	27–30	5769
10.4081/xeno.2015.5770	31–33	5770
10.4081/xeno.2015.5771	24–26	5771
10.4081/xeno.2015.5772	34–36	5772
10.4081/xeno.2015.5773	37–39	5773
10.4081/xeno.2015.5781	40–41	5781
10.4081/xeno.2016.5660	19–24	5660
10.4081/xeno.2016.5774	1–7	5774
10.4081/xeno.2016.5889	8–13	5889
10.4081/xeno.2016.6092	14–18	6092
10.4081/xeno.2016.6585	25–27	6585
10.4081/xeno.2016.6586	28–32	6586
10.4081/xeno.2016.6587	33–35	6587
10.4081/xeno.2016.6588	36–38	6588
10.4081/xeno.2016.6589	39–40	6589
10.4081/xeno.2016.6721	41–44	6721
10.4081/xeno.2017.6475	1–9	6475
10.4081/xeno.2017.6702	10–14	6702
10.4081/xeno.2017.7101	15–20	7101
10.4081/xeno.2017.7149	25–30	7149
10.4081/xeno.2017.7173	21–24	7173
10.4081/xeno.2018.7674	1–3	7674
10.4081/xeno.2018.7809	4–6	7809
10.4081/xeno.2018.7810	10–12	7810
10.4081/xeno.2018.7820	7–9	7820
10.4081/xeno.2019.8147	1–3	8147

**Table 2:** MDPI’s corrected Issue Numbers for 14 articles in Volume 3.

DOI	Previous Issue Number	Current Issue Number
10.4081/xeno.2013.s1.e1	11	s1
10.4081/xeno.2013.s1.e2	11	s1
10.4081/xeno.2013.s1.e3	11	s1
10.4081/xeno.2013.s1.e4	11	s1
10.4081/xeno.2013.s1.e5	11	s1
10.4081/xeno.2013.s1.e6	11	s1
10.4081/xeno.2013.s1.e7	11	s1
10.4081/xeno.2013.s1.e8	11	s1
10.4081/xeno.2013.s1.e9	11	s1
10.4081/xeno.2013.s1.e10	11	s1
10.4081/xeno.2013.s1.e11	11	s1
10.4081/xeno.2013.s1.e12	11	s1
10.4081/xeno.2013.s1.e13	11	s1
10.4081/xeno.2013.s1.e14	11	s1
